# Noninvasive Immunometabolic Cardiac Inflammation Imaging Using Hyperpolarized Magnetic Resonance

**DOI:** 10.1161/CIRCRESAHA.117.312535

**Published:** 2018-04-12

**Authors:** Andrew J.M. Lewis, Jack J. Miller, Angus Z. Lau, Mary K. Curtis, Oliver J. Rider, Robin P. Choudhury, Stefan Neubauer, Charles H. Cunningham, Carolyn A. Carr, Damian J. Tyler

**Affiliations:** From the Department of Physiology, Anatomy, and Genetics (A.J.M.L., J.J.M., M.K.C., C.A.C., D.J.T.), Department of Physics, Clarendon Laboratory (J.J.M.), Radcliffe Department of Medicine (A.J.M.L., O.J.R., R.P.C., S.N.), and Acute Vascular Imaging Centre (R.P.C.), Radcliffe Department of Medicine, University of Oxford, United Kingdom; and Department of Medical Biophysics, University of Toronto, Ontario, Canada (A.Z.L., C.H.C.).

**Keywords:** animals, cell line, magnetic resonance imaging, monocytes, myocardial infarction

## Abstract

**Rationale:**

Current cardiovascular clinical imaging techniques offer only limited assessment of innate immune cell-driven inflammation, which is a potential therapeutic target in myocardial infarction (MI) and other diseases. Hyperpolarized magnetic resonance (MR) is an emerging imaging technology that generates contrast agents with 10- to 20 000-fold improvements in MR signal, enabling cardiac metabolite mapping.

**Objective:**

To determine whether hyperpolarized MR using [1-^13^C]pyruvate can assess the local cardiac inflammatory response after MI.

**Methods and Results:**

We performed hyperpolarized [1-^13^C]pyruvate MR studies in small and large animal models of MI and in macrophage-like cell lines and measured the resulting [1-^13^C]lactate signals. MI caused intense [1-^13^C]lactate signal in healing myocardial segments at both day 3 and 7 after rodent MI, which was normalized at both time points after monocyte/macrophage depletion. A near-identical [1-^13^C]lactate signature was also seen at day 7 after experimental MI in pigs. Hyperpolarized [1-^13^C]pyruvate MR spectroscopy in macrophage-like cell suspensions demonstrated that macrophage activation and polarization with lipopolysaccharide almost doubled hyperpolarized lactate label flux rates in vitro; blockade of glycolysis with 2-deoxyglucose in activated cells normalized lactate label flux rates and markedly inhibited the production of key proinflammatory cytokines. Systemic administration of 2-deoxyglucose after rodent MI normalized the hyperpolarized [1-^13^C]lactate signal in healing myocardial segments at day 3 and also caused dose-dependent improvement in IL (interleukin)-1β expression in infarct tissue without impairing the production of key reparative cytokines. Cine MRI demonstrated improvements in systolic function in 2-DG (2-deoxyglucose)–treated rats at 3 months.

**Conclusions:**

Hyperpolarized MR using [1-^13^C]pyruvate provides a novel method for the assessment of innate immune cell-driven inflammation in the heart after MI, with broad potential applicability across other cardiovascular disease states and suitability for early clinical translation.

## Abstract 2

Supplemental Digital Content is available in the text.

Magnetic resonance (MR) provides exquisite structural imaging of the cardiovascular system, although current proton-based techniques provide only limited assessment of cellular disease processes, including inflammation. Innate immune cells are now understood to be pathophysiological mediators and potential therapeutic targets in myocardial infarction (MI), atherosclerosis, and other cardiovascular diseases.^1,2^ However, clinical translation of a greatly improved understanding of the biology and pathophysiological mechanisms of innate immune cells after MI^3,4^ has been limited, in part, because of the absence of imaging tools to define the monocyte/macrophage response in humans.

**Editorial, see p 1039**

**In This Issue, see p 1033**

**Meet the First Author, see p 1034**

Hyperpolarized MR is an emerging technology in which the magnetic properties of external molecules are manipulated to create molecular MR contrast agents with improvements in signal-to-noise ratio of several orders of magnitude.^5^ One key hyperpolarized substrate, [1-^13^C]pyruvate, shows particular promise for cardiovascular applications^6,7^ and enables the detection of its downstream metabolic products, lactate and bicarbonate, at millimolar concentration with spatial localization within an organ of interest.^8^ Hyperpolarized [1-^13^C]pyruvate has recently been administered to healthy human volunteers in pilot studies with sufficient signal-to-noise ratio to enable cardiac metabolite mapping with high spatial and temporal resolution using multinuclear clinical MRI systems.^9^

Most experimental cardiovascular applications of hyperpolarized MR have to date been conducted with the intention of studying cardiomyocyte metabolism; however, because the monocarboxylate transporters responsible for the cellular uptake of [1-^13^C]pyruvate are widely expressed,^10^ hyperpolarized substrates could in principle also be used to assess metabolically active, noncardiomyocyte cell populations within hearts, such as innate immune cells. Furthermore, because hyperpolarized [1-^13^C]pyruvate MR uniquely assesses 13C label flux through both LDH (lactate dehydrogenase) and PDH (pyruvate dehydrogenase), it holds potential to separate these cell populations by exploiting the intrinsic differences in cellular metabolic machinery and phenotype between immune cells (which are predominantly glycolytic^11^) and cardiomyocytes (predominantly oxidative^12^). Hyperpolarized MR, therefore, has the potential to overcome the insensitivity of existing cardiac inflammation imaging techniques and has other potential advantages, including the absence of ionizing radiation, rapid acquisition times, and near-simultaneous, high-resolution, anatomic and functional imaging with accurate metabolite coregistration.

Here, we show that hyperpolarized [1-^13^C]pyruvate MR imaging and spectroscopy provide a new approach for imaging the presence and activity of innate immune cells in myocardial tissue by exploiting metabolic reprogramming toward glycolysis in activated monocytes/macrophages, which results in a high [1-^13^C]lactate signature. The underlying metabolic reprogramming is essential for monocyte/macrophage inflammatory function, enabling MR-visible pharmacological immunomodulation of the local myocardial inflammatory response to MI.

## Methods

The study data are available on reasonable request.

Full details of the experimental methods used, including hyperpolarized MRI, flow cytometry, cell culture, quantitative polymerase chain reaction, and ELISA, are described in the Online Data Supplement.

### Statistical Analysis

Data are presented as mean±SEM; statistical comparisons are identified in the figures. In brief, where 3 groups were compared, statistical comparisons were by 1-way ANOVA with the Holm–Sidak correction for multiple comparisons, and where 2 experimental groups were compared, statistical comparisons were performed using either paired or unpaired *t* tests as appropriate.

## Results

### High Hyperpolarized [1-^13^C]Lactate Signal at Days 3 and 7 Post-MI Is Because of Monocyte/Macrophage-Driven Inflammation

MI is known to induce an intense local cardiac inflammatory response, including biphasic monocyte/macrophage accumulation.^13^ After an influx of monocytes with a primarily inflammatory phenotype (which peak in number at day 3), the myocardium subsequently recruits monocytes with broadly reparative functions (which are the dominant population by day 7^13^). To determine whether these processes could be assessed using hyperpolarized MRI, we performed either experimental cryoinfarction or a sham procedure in rats before hyperpolarized MRI, cine MRI, and heart isolation for flow cytometric analysis at day 3 or 7 after surgery (n=6 rats per time point per group). The custom-designed spectral spatial echo-planar imaging sequence provides 3-dimensional cardiac metabolite mapping with a spatial resolution of 2×2×3.8 mm^3^ and temporal resolution of 1.8 seconds.^14^ [^13^C]lactate signals were measured from segmentation regions of interest drawn manually around the infarct region (identified as the hypokinetic segments on cine MR) and the spatially average lactate signal normalized by mask size, flip angle, and whole-heart pyruvate signal.

**Figure 1.**
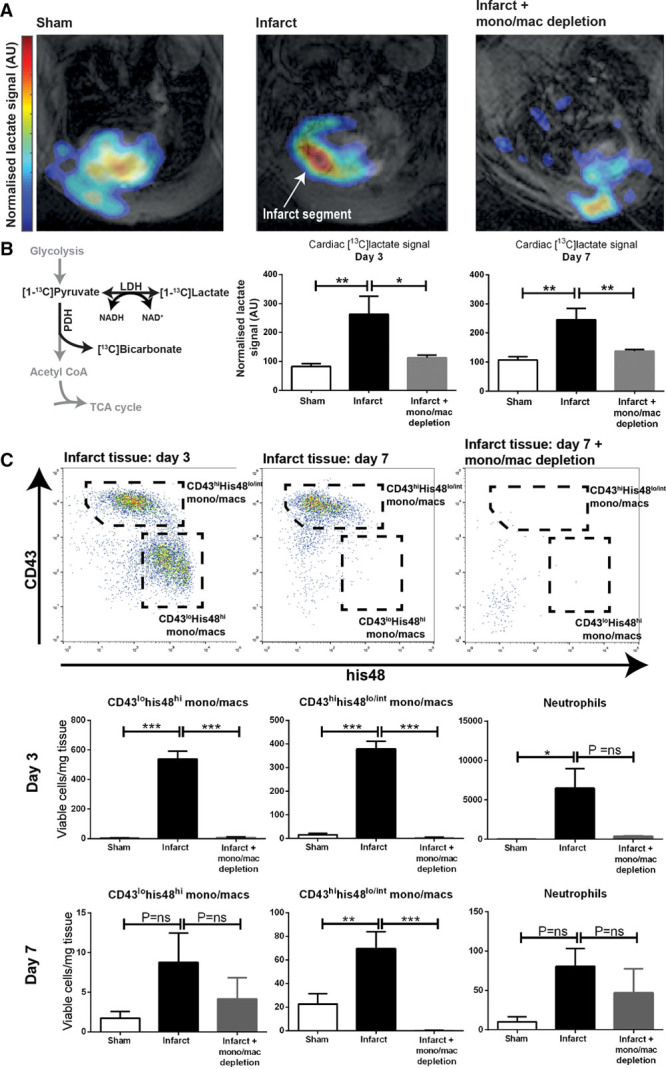
**Hyperpolarized lactate generation in a rodent model of cryoinfarction. A** and **B**, Hyperpolarized MRI demonstrated intense [1-^13^C]lactate signal at both days 3 and 7 post-experimental myocardial infarction, which was normalized in rats undergoing pharmacological macrophage depletion (n=6 rats assigned per time point per group [n= 36] to give 4–6 evaluable datasets per time point per group; mean±SEM; statistical comparison by 1-way ANOVA with the Holm–Sidak correction for multiple comparisons). Higher lactate signal is detected from the anterior portion of the heart because of some inhomogeneity from the 13C surface coil; lactate signal detected from the chest wall may reflect inflammation in the healing incision. **C**, Flow cytometric analysis of infarct tissue confirmed predominantly inflammatory monocyte/macrophage phenotype (CD43^lo^his48^hi^) at day 3 and predominantly reparative (CD43^hi^his48^lo/int^) phenotype at day 7 and that both subsets were depleted after the administration of clodronate liposomes. *P≤0.05, **P≤0.01, ***P≤0.001. LDH indicates lactate dehydrogenase; NADH, nicotinamide adenine dinucleotide; PDH, pyruvate dehydrogenase; and TCA, tricarboxylic acid.

We identified intense [1-^13^C]lactate signal in the hypokinetic, healing infarct segments in the separate groups of rats imaged at either day 3 or 7 (Figure 1A and 1B). Quantitative analysis of the lactate signal from infarct regions of interest confirmed a >2-fold increase in [1-^13^C]lactate signal when compared with sham-operated controls (*P*<0.01). In pilot studies, the same high [^13^C]lactate signature was observed with both coronary artery ligation and cryoinfarction models of MI in rats, although cryoinfarction led to more reproducible infarct sizes and lower mortality and was, therefore, used for the remainder of the study.

Rat hearts were isolated immediately after the MRI assessments for enzymatic digestion and flow cytometric analysis. This confirmed that the high [1-^13^C]lactate signature was associated with high numbers of monocytes/macrophages relative to sham-operated controls (Figure 1C). These monocytes/macrophages were as expected predominantly of inflammatory phenotype (CD43^lo^his48^hi^) at day 3 and reparative phenotype (CD43^hi^his48^lo/int^) at day 7 because CD43 expression in rat monocytes/macrophages exhibits reciprocal expression to the murine Ly6C marker.^15^

To mechanistically test the contribution of the monocyte/macrophage population to the [1-^13^C]lactate signature, we next performed monocyte/macrophage depletion by administering clodronate liposomes to rats after cryoinfarction (n=6 at each time point). Efficient depletion of the monocyte/macrophage population was confirmed by flow cytometry with a 99% reduction in CD43^lo^his48^hi^ monocytes/macrophages at day 3 (Figure 1C). Equivalent myocardial injury sizes were confirmed by cine MRI (Online Figure II) with identical increases in end-systolic volume and decreases in ejection fraction seen in both groups relative to sham controls. Monocyte/macrophage depletion with clodronate liposomes essentially normalized the [1-^13^C]lactate signal recorded at both time points (*P*<0.05 at day 3 and *P*<0.01 at day 7). These findings suggest that monocytes/macrophages are responsible for the [1-^13^C]lactate signature detected by hyperpolarized MRI and that hyperpolarized MRI is, therefore, sensitive to the post-MI monocytes/macrophage inflammatory response, although it does not differentiate the 2 phases of the response.

We next asked whether a similar effect would also be present in a more clinically relevant large animal model of MI. To test this, and to provide cross-species validation, we performed hyperpolarized [1-^13^C]pyruvate MR imaging at baseline and 7 days after coronary artery balloon-occlusion MI in pigs (n=7). The imaging sequence for these porcine experiments provides whole-heart coverage with spatial resolution of 10.7×10.7 mm^2^ for 6 slices covering the left ventricle from base to apex. The [^13^C]lactate signal was derived from the apical slices covering the infarct region and normalized to [1-^13^C]pyruvate signal intensity.

**Figure 2.**
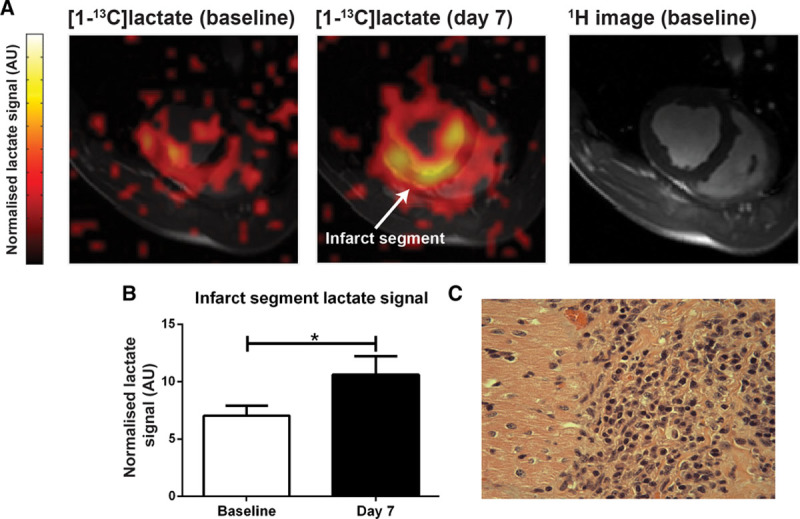
**Hyperpolarized lactate generation in a porcine model of myocardial infarction. A** and **B**, High hyperpolarized lactate signal was again detected in infarct tissue at day 7 in a large animal model of myocardial infarction when compared with the same animal pre-infarction (n=7; mean±SEM; statistical comparison by paired 2-tailed *t* test). **C**, The high lactate signal again corresponded to macrophage accumulation on histopathologic examination of the tissue. *P≤0.05, **P≤0.01, ***P≤0.001.

Again, high [1-^13^C]lactate signal was observed in infarct segments at day 7 (Figure 2A), with a 45% increase in [1-^13^C]lactate signal when compared with baseline (*P*<0.05; Figure 2A and 2B). No significant difference in [1-^13^C]bicarbonate signal was detected. Histopathologic examination of these pig hearts confirmed that the regions of highest [1-^13^C]lactate signal corresponded to high numbers of macrophages (Figure 2C), consistent with the rodent experiments.

### Metabolic Reprogramming in Activated Macrophages Causes High Hyperpolarized [1-^13^C]Lactate Signal and Is Essential for Proinflammatory Macrophage Function

The hypoxic microenvironment of infarcted myocardium would be expected to provide a significant cellular energetic challenge to infiltrating immune cells, which have a myriad of energetically demanding roles, including proteolysis, phagocytosis, and collagen deposition.^16,17^ Because glycolysis is an oxygen-efficient pathway for ATP synthesis, it follows that high [1-^13^C]lactate signal detected in vivo post-MI might reflect metabolic reprogramming in monocytes/macrophages activated by immunologic danger signals released from the necrotic myocardium. To test this, we used an 11.7T vertical bore MR system to measure hyperpolarized [1-^13^C]lactate signals spectroscopically from a controlled number of macrophages in suspension. We selected the RAW264.7 macrophage cell line to generate the high numbers of cells required for robust analysis of these data, including kinetic modeling (8×10^7^ cells per biological replicate, n=6 biological replicates per group).

**Figure 3.**
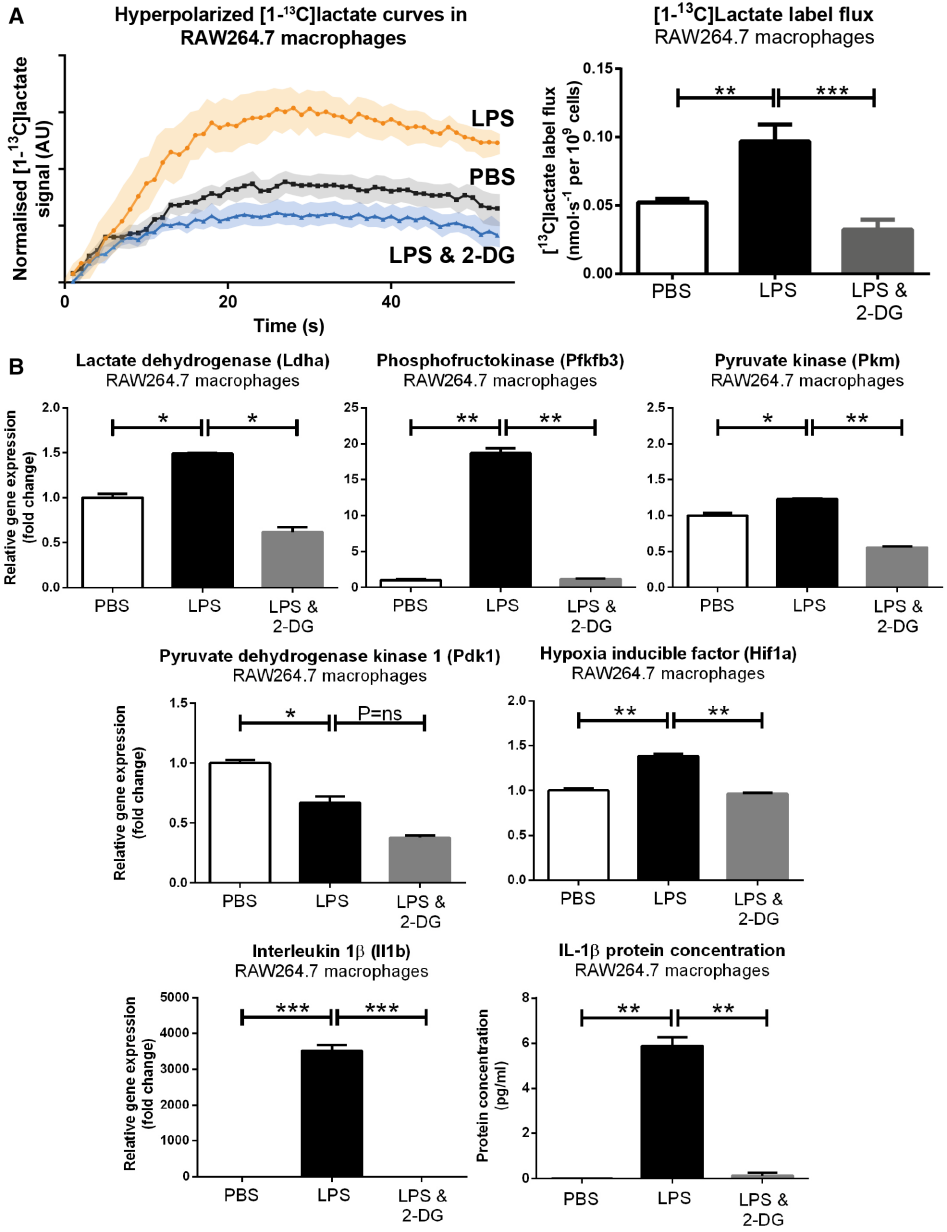
**Hyperpolarized lactate generation in activated RAW264.7 cell suspensions. A**, Hyperpolarized MR experiments in RAW264.7 cell suspensions demonstrated high lactate signal and label flux rates in activated cells, which was normalized by 2-DG (2-deoxyglucose; 80×10^6^ cells per biological replicate; mean±SEM; n=5–6 evaluable datasets per group; 1-way ANOVA with the Holm–Sidak correction for multiple comparisons; PBS; lipopolysaccharide [LPS]). [^13^C]lactate signal intensity curves (normalized to [1-^13^C]pyruvate signal) after [1-^13^C]pyruvate injection are presented; shaded region denotes SEM. [^13^C]lactate label flux rates were derived by kinetic modeling of the spectroscopic data multiplied by the final pyruvate concentration in solution. **B**, LPS caused regulation of a panel of genes encoding glycolytic enzymes and proinflammatory cytokines, which were attenuated after administration of 2-DG. Similar patterns of gene regulation were demonstrated in primary murine spleen-derived macrophages (Online Figure II). *P≤0.05, **P≤0.01, ***P≤0.001. IL indicates interleukin.

We found that polarization of RAW264.7 macrophages using lipopolysaccharide strikingly increased the raw [1-^13^C]lactate signal detected spectroscopically from a given number of cells when compared with the same number of saline-treated quiescent cells (Figure 3A). We used kinetic modeling of the dynamic spectroscopic data to estimate pyruvate to lactate exchange constants^18^ and multiplied these by the final pyruvate concentration in solution to derive cellular [^13^C]lactate label flux rates. This analysis confirmed that [^13^C]lactate label flux rates were approximately doubled in activated macrophages (*P*<0.01 when compared with saline-treated control cells; Figure 3A), suggesting that both the influx of monocytes/macrophages and also metabolic reprogramming in these activated cells may contribute to the high lactate signature detected after MI in vivo.

Gene expression analysis of RAW264.7 cells using quantitative polymerase chain reaction confirmed that polarization with lipopolysaccharide caused regulation of a panel of genes encoding key glycolytic enzymes, including LDH, PKM (pyruvate kinase), and PFKFB3 (6-phosphofructo-2-kinase/fructose-2,6-biphosphatase3; Figure 3B), alongside key proinflammatory cytokines, including IL (interleukin)-1β. These findings suggest that transcriptionally mediated metabolic reprogramming in activated RAW264.7 macrophage-like cells leads to higher glycolytic rates, providing a potential mechanism for the high [1-^13^C]lactate signal and flux rates detected. PDK1 (PDH kinase 1) is a negative regulator of PDH activity and was downregulated, which, therefore, implies an increased PDH flux. This finding suggests that the increase in cellular glycolytic rate is accompanied by an increase in glucose oxidation via PDH, although this was not to a sufficient degree to cause a detectable hyperpolarized [1-^13^C]bicarbonate signal (which reflects PDH flux) in this experiment.

A near-identical pattern of gene regulation was demonstrated in primary murine spleen-derived macrophages, performed to validate these cell line experiments (Online Figure III).

To further understand the relationship between lipopolysaccharide-induced macrophage metabolic reprogramming, hyperpolarized [1-^13^C]lactate signal, and inflammatory cytokine synthesis, we next tested the effect of coincubation of lipopolysaccharide-treated RAW264.7 cells with 2-deoxyglucose, which blocks glycolysis at the level of the hexokinase reaction and stabilizes hypoxia-inducible factor 1α signaling.^19^ We found that 2-DG (2-deoxyglucose) treatment normalized the RAW264.7 macrophage-hyperpolarized [1-^13^C]lactate signal (*P*<0.01) by inhibiting lipopolysaccharide-mediated transcriptional metabolic reprogramming and abrogating the upregulation of LDH, PKM, and PFKFB3 (Figure 3B). 2-DG also strikingly inhibited the synthesis of IL-1β (which has been reported previously^19,20^), highlighting a link between hyperpolarized [1-^13^C]lactate signal and cellular inflammatory function in vitro.

### MR-Visible Metabolic Immunomodulation Improves Cardiac Systolic Function Post-MI

We next sought to test the links between hyperpolarized [1-^13^C]lactate signal, the macrophage response for MI, and the resulting effects on cardiac remodeling and systolic function in vivo, with the aim of understanding whether hyperpolarized MR may have a potential future role as an imaging biomarker to test immunomodulation after MI. Although nonselective immunosuppression in this setting is known to be associated with impaired cardiac wound healing and a risk of ventricular rupture,^21,22^ selective targeting of the phase 1 monocyte/macrophage proinflammatory response pathways may be a viable strategy to improve cardiac remodeling and systolic function.^23,24^

**Figure 4.**
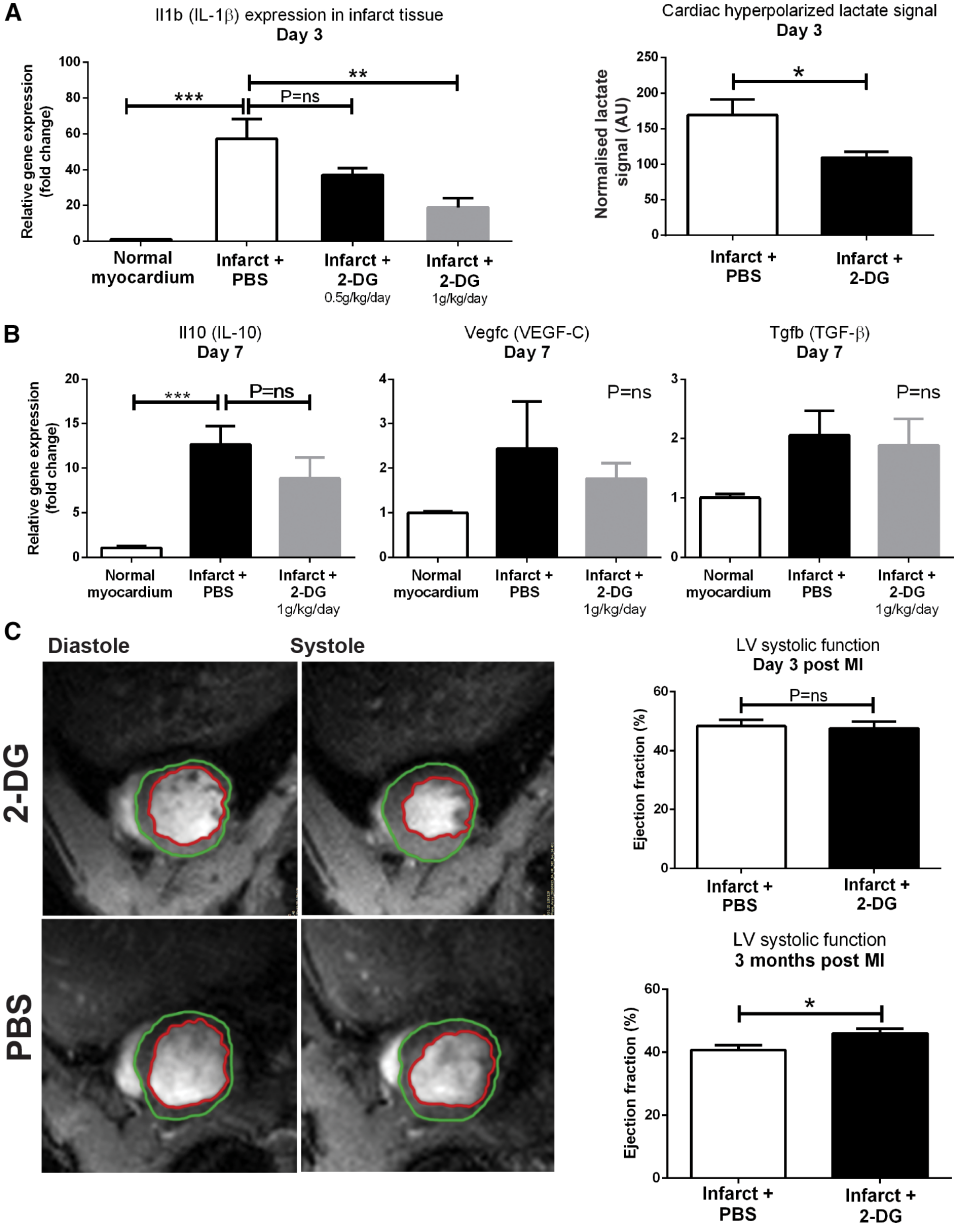
**Systemic administration of 2-DG (2-deoxyglucose) to rats post-myocardial infarction (MI) caused dose-dependent downregulation of IL (interleukin)-1β gene expression in infarct tissue and also normalized hyperpolarized [1-^13^C]lactate signal (n=4–5 evaluable datasets per group).** 2-DG had no significant effect on expression of genes encoding the proreparative factors IL-10, VEGF-C (vascular endothelial growth factor C), and TGF-β (transforming growth factor-β; n=3–4 biological replicates per group; 1-way ANOVA with the Holm–Sidak correction for multiple comparisons) Cine MRI analysis demonstrated that 2-DG attenuated the rate of decline in left ventricle (LV) systolic function between day 3 and 3 months (n=9 biological replicates per group to give 7–9 evaluable data sets; unpaired unequal variance *t* test). *P≤0.05, **P≤0.01, ***P≤0.001.

To address this, we administered either 2-DG or saline to rats between days 0 and 3 after cryoinfarction (n=12). We found that 2-DG caused dose-dependent downregulation of IL-1β gene expression in infarct tissue (Figure 4A) with a 66% reduction at the higher dose of 1g/kg per day in 2 divided doses (*P*<0.01). This dose of 2-DG also reduced the high hyperpolarized [1-^13^C]lactate signal detected by hyperpolarized MR at day 3 (*P*<0.05), which was suppressed to a level similar to that observed in sham-treated animals in previous experiments. Because our previous cell depletion experiments have suggested that elevated cardiac [^13^C]lactate signal post-MI is caused by monocytes/macrophages, this finding implies sufficient delivery of 2-DG to the cardiac monocyte/macrophage population to reproduce the anti-inflammatory effect demonstrated in vitro. It furthermore highlights a mechanistic link between hyperpolarized [1-^13^C]lactate signal and cardiac tissue-level inflammation.

The 2-DG dosing strategy (0–72 hours) was selected with the intent of suppressing primarily the phase 1 inflammatory response while preserving tissue repair processes in phase 2 (maximal at day 7). To determine whether this was the case, we measured the day 7 expression of cytokines and growth factors with roles in cardiac repair, revascularization, and mature collagen deposition,^25^ including TGF-β (transforming growth factor-β), IL-10, and VEGF-C (vascular endothelial growth factor C; Figure 4B) in healthy control rats and rats undergoing cryoinfarction with either saline treatment or 2-DG treatment between days 0 and 3 (n=12). We found no significant difference in the expression of these factors. These findings suggest that this regimen of 2-DG selectively modulates phase 1 of the immune response post-MI. Consistent with this, no incidence of ventricular rupture was detected by cine MRI in any 2-DG–treated rat.

Because selective modulation of the phase 1 inflammatory response is a potential therapeutic target after MI, we next asked what effect MR-visible immunomodulation of IL-1β synthesis with 2-DG would have on cardiac remodeling and systolic function, assessed using cine MRI. Control- and 2-DG–treated rats had equivalent ventricular volumes and systolic function at day 3, which suggests equivalent acute injury size (Online Figure IV). The left ventricles in both groups dilated by around 30% between day 3 and 3 months during remodeling, and the rate of increase of end-systolic volume was modestly attenuated in 2-DG–treated rats, with a 5% absolute improvement in ejection fraction at 3 months (*P*<0.05; Figure 4C). These findings suggest that 2-DG–mediated immunomodulation post-MI can be monitored noninvasively using hyperpolarized MRI and may improve cardiac remodeling by selectively targeting the phase I inflammatory response.

## Discussion

Acute MI remains a leading cause of heart failure, despite effective reperfusion strategies.^26^ The development of new imaging technologies to define pathways, which could be targeted in the days after MI with the aim of improving cardiac remodeling and reducing heart failure, therefore, represents an important and clinically relevant goal.

Our finding that the influx and metabolic reprogramming of activated innate immune cells within infarct tissue can be assessed using hyperpolarized [1-^13^C]pyruvate MR provides an approach by which MR could be used to better understand innate immune cell biology in human cardiovascular disease. Conventional proton MR-based techniques, including T_1_ parametric mapping and T_2_ weighted imaging, are known to be insensitive to immune cell number after MI because any signal change attributable to these cells is masked by much larger changes in ^1^H T_1_ and T_2_ resulting from edema and collagen deposition.^27,28^ It follows that the combination of hyperpolarized [1-^13^C]pyruvate MR (to assess inflammation via [^13^C]lactate mapping) with ^1^H T_1_, T_2_, and gadolinium-based contrast agent techniques, including extracellular volume mapping (for tissue characterization and functional imaging) could be used to provide a more comprehensive assessment of the interplay between the myocardial inflammatory response and the resulting tissue architecture and ventricular function than is possible currently. Furthermore, because the immunologic danger signals released from necrotic myocardium polarize quiescent innate immune cells via their conserved pattern recognition/toll-like receptors,^29,30^ inflammation imaging with hyperpolarized [1-^13^C]pyruvate MR may well be broadly applicable across other cardiovascular disease states involving innate immune cell activation, including atherosclerosis, myocarditis, and endocarditis.

Previous studies using positron emission tomography (PET) of the 18-fluorodeoxyglucose (^18^FDG) tracer have demonstrated high cardiac^18^FDG uptake in the days after MI, which may reflect similar cellular processes to those resulting in high [1-^13^C]lactate signal in this study.^31^ However, cardiac ^18^FDG PET can be confounded by high background glucose uptake by the myocardium, and its ability to detect the smaller signal resulting from immune cell activity is, therefore, critically dependent on artificial suppression of cardiac carbohydrate metabolism using, for example, high fat/low carbohydrate diets in clinical studies, and the mode of anesthesia used in preclinical studies.^31^ The failure rate of cardiac carbohydrate uptake suppression in humans is reported to be 20% to 30%.^32^

Newer PET tracers targeting macrophage cell surface proteins (such as ^68^Ga-DOTATATE or ^68^Ga-DOTA-TOC, which bind SST_2_ [somatostatin receptor subtype-2], and ^68^Ga-citrate, which binds lactoferrin) show promise for the identification of inflammatory activity of monocytes and macrophages.^33^ Furthermore, because SST_2_ is exclusively expressed in proinflammatory M1-type macrophages, ^68^Ga-DOTATATE offers reliable imaging of inflammatory activity in active atherosclerotic plaques,^33,34^ avoiding the limitation of ^18^FDG myocardial spillover for coronary imaging. These tracers have been clinically translated to human use and in a clinical investigation, ^68^Ga-DOTA-TOC demonstrated consistent uptake in patients with myocarditis or subacute MI,^35^ as well as in a patient with cardiac involvement from sarcoidosis.^36^ Balanced against these studies, in a detailed preclinical investigation, neither ^68^Ga-DOTATATE nor ^68^Ga-citrate showed appreciable uptake in myocardial infarct tissue after MI.^37^ Further leukocyte tracers, including those targeting the chemokine receptor CXCR4 (C-X-C chemokine receptor type 4)^38^ or translocator protein,^39^ are in development although they remain to be tested post-MI. In future clinical studies, the performance of these PET tracers for post-MI inflammation imaging could be prospectively tested alongside alternative MRI technologies, including 19F MRI using emulsified perfluorocarbons,^40,41^ which shows promise in preclinical models or iron oxide nanoparticles, as well as hyperpolarized [1-^13^C]pyruvate.

Hyperpolarized [1-^13^C]pyruvate offers several advantages over ^18^FDG for cardiac applications. First, the ability to image the downstream products of glucose metabolism offers the potential to improve specificity when compared to measurement of glucose uptake because of the divergent metabolic phenotypes of glycolytic immune cells and oxidative cardiomyocytes. Furthermore, hyperpolarized MR enables imaging without the need for artificial suppression of cardiomyocyte metabolism, the absence of ionizing radiation (enabling longitudinal studies in humans), faster acquisition times (cardiac hyperpolarized MR acquisitions typically last 2–5 minutes compared with cardiac gated PET acquisitions, which can take >30 minutes), and the ability to normalize the [1-^13^C]lactate signal to tissue delivery rates of the [1-^13^C]pyruvate substrate, which may be particularly important after MI when tissue delivery rates of the tracer may be impaired by either an epicardial coronary stenosis or microvascular dysfunction. Furthermore, because lactate is increasingly recognized to be an immunologic danger signal in its own right,^20^ assessment of the lactate pool size might provide a more fundamental assessment of innate immune cell immunometabolic function after MI than glucose uptake alone. It is, however, not yet clear whether clinical cardiac hyperpolarized MRI will achieve or exceed the spatial resolution of PET (which is typically 4–6 mm for ^18^FDG^42^) because clinical 13C MR coils and sequences are still in their infancy. Furthermore, clinical grade hyperpolarizers are currently only available in a few centers worldwide, unlike PET cyclotrons and scanners.

IL-1β is a cytokine known to control processes determining myocardial remodeling post-MI,^23,43,44^ and most therapeutic studies to date have focussed on downstream targeting of the cytokine using monoclonal antibodies. Although this approach avoids the potentially deleterious effects of nonselective immunosuppression with, for example, corticosteroids, the lack of routine availability of human myocardial tissue for cytokine assay or assessment of inflammation post-MI limits the potential for individualization of therapy or validation of dosing strategy.^45^ Given that the degree of myocardial injury and subsequent inflammation after acute coronary syndromes is highly heterogeneous, it is perhaps not surprising that clinical trials involving largely unselected patients with MI have had variable results to date.^46–48^ Hyperpolarized MR may, therefore, have an early role in the identification of patient subgroups with the highest degree of myocardial inflammation, who might be expected to derive the most benefit from currently available immunomodulatory therapies, such as anakinra or canakinumab. In the future, hyperpolarized MR may also have utility in the development of drugs targeting upstream pathways responsible for proinflammatory cytokines by immune cells. Although 2-deoxyglucose experiences unpredictable pharmacokinetics and toxicity and is unlikely to be tested in humans with MI,^49^ newer immunometabolic modulators are in advancing stages of development and may provide new classes of drugs for immunomodulation.^50^

In summary, hyperpolarized MR using [1-^13^C]pyruvate provides a novel noninvasive assessment of cardiac innate immune cell-driven inflammation by detecting [1-^13^C]lactate signal resulting from induction of an immunometabolic signaling axis, which is essential for inflammatory cytokine production and controls cardiac remodeling. In addition to potential future applications in human ischemic heart disease, this technique may have broad value in other forms of inflammatory disease with important advantages over existing techniques.

## Sources of Funding

This research was supported by the British Heart Foundation Centre of Research Excellence (Oxford), the National Institute for Health Research Oxford Biomedical Research Centre Programme, the British Heart Foundation Fellowship (FS/10/002/28078 and FS/14/17/30634), the British Heart Foundation Programme Grant (RG/11/9/28921), and the Engineering and Physical Sciences Research Council Doctoral Training Centre and Prize Fellowship (EP/M508111/1) grants. R.P. Choudhury was supported by the British Heart Foundation Centre of Research Excellence (Oxford), the British Heart Foundation Oxbridge Centre for Regenerative Medicine, and the Tripartite Immunometabolism Consortium–Novo Nordisk Foundation (grant No. NNF15CC0018486).

## Disclosures

None.

## Acknowledgments

A.J.M. Lewis conceived the experiment, conducted experimental work, analyzed data, and drafted the manuscript. J.J. Miller designed MR imaging sequence and methodology for data analysis and performed rodent hyperpolarized MRI experiments. A.Z. Lau designed MR imaging sequence and methodology for data analysis and performed porcine hyperpolarized MRI and infarction experiments. M.K. Curtis performed experimental work. C.A. Carr, O.J. Rider, R.P. Choudhury, S. Neubauer, and D.J. Tyler were responsible for resources and supervised the preclinical experiments. All authors reviewed and commented on the manuscript.

## Novelty and Significance

### What Is Known?

- The myocardial inflammatory response that follows myocardial infarction (MI) may be a future clinical therapeutic target.
- There is a lack of clinical imaging tools to assess the cardiac monocyte/macrophage response noninvasively.
- Hyperpolarized magnetic resonance is an emerging imaging technology in which the magnetic resonance imaging signal of key substrates is enhanced by several orders of magnitude, enabling noninvasive cardiac metabolite mapping.

## References

[1] Swirski FK, Nahrendorf M (2013). Leukocyte behavior in atherosclerosis, myocardial infarction, and heart failure.. Science.

[2] Frangogiannis NG (2014). The inflammatory response in myocardial injury, repair, and remodelling.. Nat Rev Cardiol.

[3] Swirski FK, Nahrendorf M, Etzrodt M, Wildgruber M, Cortez-Retamozo V, Panizzi P, Figueiredo JL, Kohler RH, Chudnovskiy A, Waterman P, Aikawa E, Mempel TR, Libby P, Weissleder R, Pittet MJ (2009). Identification of splenic reservoir monocytes and their deployment to inflammatory sites.. Science.

[4] Dutta P, Courties G, Wei Y (2012). Myocardial infarction accelerates atherosclerosis.. Nature.

[5] Ardenkjær-Larsen JH, Fridlund B, Gram A, Hansson G, Hansson L, Lerche MH, Servin R, Thaning M, Golman K (2003). Increase in signal-to-noise ratio of > 10,000 times in liquid-state NMR.. Proc Natl Acad Sci USA.

[6] Day SE, Kettunen MI, Gallagher FA, Hu DE, Lerche M, Wolber J, Golman K, Ardenkjaer-Larsen JH, Brindle KM (2007). Detecting tumor response to treatment using hyperpolarized 13C magnetic resonance imaging and spectroscopy.. Nat Med.

[7] Schroeder MA, Cochlin LE, Heather LC, Clarke K, Radda GK, Tyler DJ (2008). In vivo assessment of pyruvate dehydrogenase flux in the heart using hyperpolarized carbon-13 magnetic resonance.. Proc Natl Acad Sci USA.

[8] Schroeder MA, Clarke K, Neubauer S, Tyler DJ (2011). Hyperpolarized magnetic resonance: a novel technique for the in vivo assessment of cardiovascular disease.. Circulation.

[9] Cunningham CH, Lau JY, Chen AP, Geraghty BJ, Perks WJ, Roifman I, Wright GA, Connelly KA (2016). Hyperpolarized 13C metabolic MRI of the human heart: initial experience.. Circ Res.

[10] Enerson BE, Drewes LR (2003). Molecular features, regulation, and function of monocarboxylate transporters: implications for drug delivery.. J Pharm Sci.

[11] Kelly B, O’Neill LA (2015). Metabolic reprogramming in macrophages and dendritic cells in innate immunity.. Cell Res.

[12] Neubauer S (2007). The failing heart–an engine out of fuel.. N Engl J Med.

[13] Nahrendorf M, Swirski FK, Aikawa E, Stangenberg L, Wurdinger T, Figueiredo JL, Libby P, Weissleder R, Pittet MJ (2007). The healing myocardium sequentially mobilizes two monocyte subsets with divergent and complementary functions.. J Exp Med.

[14] Miller JJ, Lau AZ, Teh I, Schneider JE, Kinchesh P, Smart S, Ball V, Sibson NR, Tyler DJ (2016). Robust and high resolution hyperpolarized metabolic imaging of the rat heart at 7 T with 3D spectral-spatial EPI.. Magn Reson Med.

[15] Barnett-Vanes A, Sharrock A, Birrell MA, Rankin S (2016). A single 9-colour flow cytometric method to characterise major leukocyte populations in the rat: validation in a model of LPS-induced pulmonary inflammation.. PLoS One.

[16] Nahrendorf M, Swirski FK (2013). Monocyte and macrophage heterogeneity in the heart.. Circ Res.

[17] Frangogiannis NG (2008). The immune system and cardiac repair.. Pharmacol Res.

[18] Zierhut ML, Yen YF, Chen AP, Bok R, Albers MJ, Zhang V, Tropp J, Park I, Vigneron DB, Kurhanewicz J, Hurd RE, Nelson SJ (2010). Kinetic modeling of hyperpolarized 13C1-pyruvate metabolism in normal rats and TRAMP mice.. J Magn Reson.

[19] Tannahill GM, Curtis AM, Adamik J (2013). Succinate is an inflammatory signal that induces IL-1β through HIF-1α.. Nature.

[20] Yang L, Xie M, Yang M, Yu Y, Zhu S, Hou W, Kang R, Lotze MT, Billiar TR, Wang H, Cao L, Tang D (2014). PKM2 regulates the Warburg effect and promotes HMGB1 release in sepsis.. Nat Commun.

[21] Roberts R, DeMello V, Sobel BE (1976). Deleterious effects of methylprednisolone in patients with myocardial infarction.. Circulation.

[22] Bulkley BH, Roberts WC (1974). Steroid therapy during acute myocardial infarction. A cause of delayed healing and of ventricular aneurysm.. Am J Med.

[23] Leuschner F, Dutta P, Gorbatov R (2011). Therapeutic siRNA silencing in inflammatory monocytes in mice.. Nat Biotechnol.

[24] Ruparelia N, Chai JT, Fisher EA, Choudhury RP (2017). Inflammatory processes in cardiovascular disease: a route to targeted therapies.. Nat Rev Cardiol.

[25] Frangogiannis NG, Mendoza LH, Lewallen M, Michael LH, Smith CW, Entman ML (2001). Induction and suppression of interferon-inducible protein 10 in reperfused myocardial infarcts may regulate angiogenesis.. FASEB J.

[26] Gerber Y, Weston SA, Enriquez-Sarano M, Manemann SM, Chamberlain AM, Jiang R, Roger VL (2016). Atherosclerotic burden and heart failure after myocardial infarction.. JAMA Cardiol.

[27] Fernández-Jiménez R, García-Prieto J, Sánchez-González J, Agüero J, López-Martín GJ, Galán-Arriola C, Molina-Iracheta A, Doohan R, Fuster V, Ibáñez B (2015). Pathophysiology underlying the bimodal edema phenomenon after myocardial ischemia/reperfusion.. J Am Coll Cardiol.

[28] Fernández-Jiménez R, Sánchez-González J, Agüero J (2015). Myocardial edema after ischemia/reperfusion is not stable and follows a bimodal pattern: imaging and histological tissue characterization.. J Am Coll Cardiol.

[29] Shishido T, Nozaki N, Yamaguchi S, Shibata Y, Nitobe J, Miyamoto T, Takahashi H, Arimoto T, Maeda K, Yamakawa M, Takeuchi O, Akira S, Takeishi Y, Kubota I (2003). Toll-like receptor-2 modulates ventricular remodeling after myocardial infarction.. Circulation.

[30] Oyama J, Blais C, Liu X, Pu M, Kobzik L, Kelly RA, Bourcier T (2004). Reduced myocardial ischemia-reperfusion injury in toll-like receptor 4-deficient mice.. Circulation.

[31] Lee WW, Marinelli B, van der Laan AM (2012). PET/MRI of inflammation in myocardial infarction.. J Am Coll Cardiol.

[32] Dweck MR, Aikawa E, Newby DE, Tarkin JM, Rudd JH, Narula J, Fayad ZA (2016). Noninvasive molecular imaging of disease activity in atherosclerosis.. Circ Res.

[33] Li X, Bauer W, Kreissl MC, Weirather J, Bauer E, Israel I, Richter D, Riehl G, Buck A, Samnick S (2013). Specific somatostatin receptor II expression in arterial plaque: (68)Ga-DOTATATE autoradiographic, immunohistochemical and flow cytometric studies in apoE-deficient mice.. Atherosclerosis.

[34] Tarkin JM, Joshi FR, Evans NR (2017). Detection of Atherosclerotic Inflammation by 68Ga-DOTATATE PET Compared to [18F]FDG PET Imaging.. J Am Coll Cardiol.

[35] Lapa C, Reiter T, Li X, Werner RA, Samnick S, Jahns R, Buck AK, Ertl G, Bauer WR (2015). Imaging of myocardial inflammation with somatostatin receptor based PET/CT - A comparison to cardiac MRI.. Int J Cardiol.

[36] Reiter T, Werner RA, Bauer WR, Lapa C (2015). Detection of cardiac sarcoidosis by macrophage-directed somatostatin receptor 2-based positron emission tomography/computed tomography.. Eur Heart J.

[37] Thackeray JT, Bankstahl JP, Wang Y, Korf-Klingebiel M, Walte A, Wittneben A, Wollert KC, Bengel FM (2015). Targeting post-infarct inflammation by PET imaging: comparison of (68)Ga-citrate and (68)Ga-DOTATATE with (18)F-FDG in a mouse model.. Eur J Nucl Med Mol Imaging.

[38] Thackeray JT, Derlin T, Haghikia A, Napp LC, Wang Y, Ross TL, Schäfer A, Tillmanns J, Wester HJ, Wollert KC, Bauersachs J, Bengel FM (2015). Molecular imaging of the chemokine receptor CXCR4 after acute myocardial infarction.. JACC Cardiovasc Imaging.

[39] Hupe HC, Thackeray J, Postema J, Wang Y, Ross TL, Wollert K, Bankstahl J, Bengel F (2016). Myocardial infarction is associated with neuroinflammation-a systemic analysis using TSPO-targeted molecular imaging.. J Nucl Med.

[40] Temme S, Jacoby C, Ding Z, Bönner F, Borg N, Schrader J, Flögel U (2014). Technical advance: monitoring the trafficking of neutrophil granulocytes and monocytes during the course of tissue inflammation by noninvasive 19F MRI.. J Leukoc Biol.

[41] Ye YX, Basse-Lüsebrink TC, Arias-Loza PA, Kocoski V, Kampf T, Gan Q, Bauer E, Sparka S, Helluy X, Hu K, Hiller KH, Boivin-Jahns V, Jakob PM, Jahns R, Bauer WR (2013). Monitoring of monocyte recruitment in reperfused myocardial infarction with intramyocardial hemorrhage and microvascular obstruction by combined fluorine 19 and proton cardiac magnetic resonance imaging.. Circulation.

[42] Rosenbaum D, Millon A, Fayad ZA (2012). Molecular imaging in atherosclerosis: FDG PET.. Curr Atheroscler Rep.

[43] Dewald O, Zymek P, Winkelmann K, Koerting A, Ren G, Abou-Khamis T, Michael LH, Rollins BJ, Entman ML, Frangogiannis NG (2005). CCL2/Monocyte Chemoattractant Protein-1 regulates inflammatory responses critical to healing myocardial infarcts.. Circ Res.

[44] Sager HB, Heidt T, Hulsmans M, Dutta P, Courties G, Sebas M, Wojtkiewicz GR, Tricot B, Iwamoto Y, Sun Y, Weissleder R, Libby P, Swirski FK, Nahrendorf M (2015). Targeting interleukin-1β reduces leukocyte production after acute myocardial infarction.. Circulation.

[45] Matthews PM, Rabiner I, Gunn R (2011). Non-invasive imaging in experimental medicine for drug development.. Curr Opin Pharmacol.

[46] Ridker PM, Thuren T, Zalewski A, Libby P (2011). Interleukin-1β inhibition and the prevention of recurrent cardiovascular events: rationale and design of the Canakinumab Anti-inflammatory Thrombosis Outcomes Study (CANTOS).. Am Heart J.

[47] O’Donoghue ML, Glaser R, Cavender MA, LATITUDE-TIMI 60 Investigators (2016). Effect of losmapimod on cardiovascular outcomes in patients hospitalized with acute myocardial infarction: a randomized clinical trial.. JAMA.

[48] Newby LK, Marber MS, Melloni C, SOLSTICE Investigators (2014). Losmapimod, a novel p38 mitogen-activated protein kinase inhibitor, in non-ST-segment elevation myocardial infarction: a randomised phase 2 trial.. Lancet.

[49] Maschek G, Savaraj N, Priebe W, Braunschweiger P, Hamilton K, Tidmarsh GF, De Young LR, Lampidis TJ (2004). 2-deoxy-D-glucose increases the efficacy of adriamycin and paclitaxel in human osteosarcoma and non-small cell lung cancers in vivo.. Cancer Res.

[50] O’Neill LA, Kishton RJ, Rathmell J (2016). A guide to immunometabolism for immunologists.. Nat Rev Immunol.

